# Exploring the Impact of Novel Anti-Cancer Therapies on Jaw Osteonecrosis and Other Bones: A Comprehensive Review

**DOI:** 10.3390/jcm13071889

**Published:** 2024-03-25

**Authors:** Wojciech Konarski, Tomasz Poboży, Klaudia Konarska, Andrzej Śliwczyński, Ireneusz Kotela, Jan Krakowiak

**Affiliations:** 1 Department of Orthopaedic Surgery, Ciechanów Hospital, 06-400 Ciechanów, Poland; tomasz.pobozy@onet.pl; 2 Medical Rehabilitation Center, Sobieskiego 47D, 05-120 Legionowo, Poland; klodiii87@gmail.com; 3 Social Medicine, Department of Social and Preventive Medicine, Medical University of Lodz, 90-647 Lodz, Poland; andrzej.sliwczynski.ahe@gmail.com (A.Ś.); jan.krakowiak@umed.lodz.pl (J.K.); 4 Department of Orthopedic Surgery and Traumatology, Central Research Hospital of Ministry of Interior, Wołoska 137, 02-507 Warsaw, Poland; ikotela@op.pl

**Keywords:** avascular necrosis, osteonecrosis, medication-related osteonecrosis of the jaw, MRONJ, tyrosine kinase inhibitors, biological treatment

## Abstract

Osteonecrosis is a debilitating condition characterized by the loss of blood supply to the bones, leading to bone death. This condition can impact various bones, including the jaw, which significantly affects patients’ quality of life by causing difficulties in swallowing, feeding, chewing, and speaking, along with swollen, painful mucous membranes and chronic sinusitis. Osteonecrosis can arise due to treatment with antiresorptive drugs. However, there is a growing number of reports of osteonecrosis following novel targeted anti-cancer treatments, such as tyrosine kinase inhibitors (TKIs) and biological therapies. The pathogenesis of osteonecrosis is linked to the side effects of the antiangiogenic mechanisms of these medications, leading to a disrupted blood flow. Our review aims to examine recent insights into osteonecrosis triggered by new anti-cancer drugs. Most reports focus on the osteonecrosis of the jaw (ONJ); however, we discovered that some authors have described cases of osteonecrosis affecting the femoral head or elbow following novel anti-cancer treatments. Prevention is a key component in managing osteonecrosis. Therefore, a comprehensive risk assessment should always be performed before and during anti-cancer therapy.

## 1. Introduction

Osteonecrosis is a condition characterized by the partial or complete loss of blood supply, resulting in bone death [1]. While osteonecrosis most frequently affects the femur, it can occur in various bones throughout the body. Osteonecrosis can be linked to a range of factors including the use of certain medications, traumatic injuries, excessive alcohol and narcotic consumption, or exposure to radiation [1,2,3]. Osteonecrosis of the jaw (ONJ), a specific form of osteonecrosis, greatly impacts the patient’s quality of life and contributes to a notable mortality rate. This condition is manifested in difficulties in swallowing, feeding, chewing, and speaking. Additionally, it is characterized by swollen, painful mucous membranes and chronic sinusitis [2,4,5]. The most severe cases can resemble the classic appearance of chronic osteomyelitis, characterized by sequestrum formation, thickening of the lamina dura, and pathological fractures [6]. In the past, ONJ was primarily associated with the use of bisphosphonates and termed bisphosphonate-related ONJ (BRONJ) [2,7]. Nitrogen-containing bisphosphonates reduce the activity of osteoclasts and slow down the process of bone remodeling. They work by blocking the action of farnesyl diphosphate synthase (FDPS), an enzyme involved in the mevalonate pathway responsible for producing cholesterol and isoprenoids. This inhibition prevents the prenylation of small GTPases, which is toxic to both osteoclasts and osteoblasts [8].

However, recent reports indicate that osteonecrosis can also result from novel targeted anti-cancer treatments, including tyrosine kinase inhibitors (TKIs) and biological treatments [9,10]. The highest level of risk is associated with vascular endothelial growth factor (VEGF) inhibitors; however, an increasing number of cases are being reported in relation to different medications [10]. Therefore, the term BRONJ has been replaced by medication-related ONJ (MRONJ) [5].

Ruggiero et al. [5] proposed a four-stage classification for MRONJ, along with the corresponding treatment strategies, as outlined in Table 1.

The range of drugs associated with an increased risk of ONJ is continuously expanding. Noteworthily, there are also reports of osteonecrosis affecting the femoral head or elbow following novel anti-cancer treatments. It is important to keep in mind that this risk is influenced by factors such as the dosage of the drug, the length of the treatment, and the presence of other medical conditions [9,11,12]. Understanding the mechanisms behind osteonecrosis caused by new cancer treatments is also crucial for improving the management of patients undergoing these therapies.

There is no specific treatment for ONJ and it mostly is based on systemic antibiotics, painkillers, mouth rinse, and surgical debridement or the resection of affected bone. The outcomes of conservative treatment, which encompasses mouth hygiene and/or antibiotic therapy, are generally unsatisfactory, with resolution of ONJ observed in 22.7% to 35% of patients [13,14]. The results from minimally invasive surgical techniques, such as debridement, show better outcomes, resulting in complete healing in 73% of patients [15]. However, prevention is key, and for optimal results, a comprehensive risk assessment should be conducted both before and during therapy [11].

Consequently, the aim of this review is to collect and analyze recent findings on osteonecrosis induced by new anti-cancer drugs, with a focus on ONJ, and to investigate whether such therapy can also cause osteonecrosis in bones other than the jaw.

## 2. Methods

We conducted searches in the PubMed and Embase databases. The keywords used in the search covered all registered TKIs and biologics in combination with term osteonecrosis. We searched keywords in titles and abstracts. An example phrase for abemaciclib was (abemaciclib [Title/Abstract]) AND (osteonecrosis [Title/Abstract]). Beyond the database search, we also reviewed the reference lists of the selected articles. We included all original reports of osteonecrosis caused by novel anti-cancer drugs, including case reports, case series, prospective and retrospective studies, and systematic reviews.

## 3. Tyrosine Kinase Inhibitors

Mutations, translocations, or amplifications leading to the abnormal activation of TKs are associated with the initiation, progression, invasion, and metastasis of cancers. Consequently, TKs have become significant targets in drug development. TKIs are a category of drugs specifically developed to disrupt the signal transduction pathways of protein kinases by employing a range of inhibitory techniques [10,16,17]. TKI therapy is commonly associated with adverse effects. The most commonly observed are cardiovascular side effects that include hypertension, atrial fibrillation, and decreased cardiac function. Additionally, there is growing evidence linking TKI therapy to osteonecrosis. Table 2 presents the characteristics of TKIs that have a confirmed association with osteonecrosis [18,19].

### 3.1. Imatinib

Imatinib acts by blocking platelet-derived growth factor receptors (PDGFRs) and c-kit, both of which play roles in angiogenesis. Imatinib can also lower VEGF expression by interfering with these pathways. Beyond its impact on blood vessels, imatinib also influences bone remodeling processes. By inhibiting signaling through c-fms and c-kit, imatinib may lead to a reduction in both the number and activity of osteoclasts [20,21,22].

In a prospective, intergroup, open-label, single-arm clinical trial focusing on pediatric patients with acute lymphoblastic leukemia treated with imatinib, 8 out of 155 patients (5%) experienced osteonecrosis. The specific bones affected were not detailed by the authors [23].

McKenna et al. [24] reported the case of a 75-year-old man who experienced bilateral ear pain and discharge for four months; he was treated with imatinib for a gastrointestinal stromal tumor. Subsequent examinations uncovered bilateral bony erosion and necrosis at the base of both external auditory canals. The patient underwent debridement to remove necrotic material and began a six-month regimen of oral ciprofloxacin, complemented by two weeks of topical gentamicin. This treatment successfully alleviated his symptoms without the need to discontinue imatinib. Okubo-Sato et al. [22] reported a case involving a 52-year-old woman who experienced exposed bone and pain upon contact in the right mandible following treatment with imatinib due to chronic myeloid leukemia (CML). She underwent sequestrectomy and had the mandibular tori removed. At the 2-year follow-up, she was stable and showed no signs of recurrence of ONJ. Gupta et al. [25] described the case of a 49-year-old male who presented with exposed bone intraorally in the right lower quadrant, specifically around the second premolar, first molar, and second molar. The patient received treatment with iodine oral rinse and an antimicrobial regimen of amoxicillin with clavulanic acid for one week. Additionally, a treatment plan, including pentoxifylline and tocopherol, was initiated to hinder the further development of ONJ. At the 8-week follow-up, the patient was in good shape and successfully resumed his daily activities. Viviano et al. [26] documented the case of a 72-year-old man who was receiving imatinib treatment for metastases from gastrointestinal stromal tumors. After 22 months of therapy, the patient developed ONJ and was treated with levofloxacin and fluconazole. It is important to highlight that none of the cases mentioned involved patients receiving concurrent treatment with bisphosphonates. However, there are reports in the literature of ONJ occurring after treatment with imatinib in combination with antiresorptive drugs [27,28].

### 3.2. Lenvatinib

Lenvatinib is a small-molecule inhibitor targeted on multi-kinases. There is the possibility of ONJ after lenvatinib treatment due to its antiangiogenic properties, which result from the inhibition of PDGF and VEGF [29].

Monteiro et al. [30] reported the case of a 61-year-old woman who experienced a painful, non-healing ulcer with bone rarefaction and exposure in the posterior right maxilla following treatment with lenvatinib for thyroid carcinoma. The patient was effectively treated through five sessions of photobiomodulation therapy. Mauceri et al. [31] also reported on a 58-year-old man who developed focal ONJ, characterized by a persisting alveolar socket and cortical disruption, following treatment with lenvatinib for thyroid carcinoma.

### 3.3. Osimertinib

Osimertinib is a third-generation EGFR-TKI, specifically targeting EGFR-activating mutations and the EGFR T790M mutation in advanced non-small cell lung cancer (NSCLC). EGFR regulates VEGF expression through the MAPK and PI3K pathways and utilizes at least three distinct transcription factors, STAT3, Sp1, and HIF-1α, independently of hypoxia conditions. Osimertinib may interfere with the EGFR pathway, potentially leading to reduced VEGF protein production and decreased angiogenesis [32,33,34]. There are two case reports of ONJ after osimertinib treatment. Wang et al. [34] reported a case where a 69-year-old woman with NSCLC developed ONJ following four years of osimertinib treatment in monotherapy. A digital volume tomography scan showed sequestrum formation. She received treatment through the surgical removal of the dead bone, along with intravenous antibiotics at the hospital. A bone biopsy and subsequent histopathological examination confirmed the diagnosis of ONJ. One-week post-operation, the patient’s wound was healing nicely without any infection signs. Subramanian et al. [35] described the case of a 75-year-old woman who experienced dental alveolar bone necrosis after a recent osimertinib treatment. The necrotic tissue was removed by curettage until the hard, bleeding bone was reached. The soft tissue was then brought together to ensure the primary closure of the treated areas. Following the debridement, the healing process was quick and without complications.

### 3.4. Sorafenib

Sorafenib, an oral multitargeted TKI, acts on the VEGF receptor family (VEGFR-2 and VEGFR-3) and the PDGFR family, both of which are crucial in tumor progression and angiogenesis. There have been reports of an association between sorafenib use and the development of osteonecrosis in some patients undergoing treatment with this medication [36]. Garuti et al. [37] described the case of a 74-year-old male diagnosed with ONJ following treatment with sorafenib for hepatocellular carcinoma. Upon surgical maxillofacial assessment, curettage treatment was not indicated as there were no evident signs of local infection or bone sequestration. Guillet et al. [38] documented a case of bilateral osteonecrosis of the femoral head in a 53-year-old man undergoing sorafenib treatment for hepatocellular carcinoma. Ten months into the treatment, the patient began experiencing hip pain during walking. Apart from pain during active and passive leg movements, the physical examination was normal. Magnetic resonance imaging (MRI) revealed bilateral osteonecrosis of the femoral heads. The report did not detail the treatment administered to this patient.

### 3.5. Sunitinib

Sunitinib inhibits the formation of new blood vessels by diminishing the activity of VEGF. VEGF also contributes to the function of osteoclast cells, and blocking this factor could shed light on the underlying mechanisms that heighten the risk of ONJ in patients treated with sunitinib [39]. There is large number of reports on ONJ after sunitinib treatment [40,41,42,43,44,45,46,47,48,49]. Vallina et al. [50] conducted a systematic review of reports involving 102 patients treated with sunitinib, of which 58 (56.86%) developed ONJ. The average age of patients afflicted with ONJ was 56 years. A majority of these patients were also on medications affecting bone remodeling, such as bisphosphonates or denosumab, alongside sunitinib. Specifically, 49 patients (84%) with ONJ received both sunitinib and bisphosphonates, while 5 patients (9%) were solely on sunitinib. In terms of ONJ’s location, it was unspecified in 8 cases (13%), with 34 cases (54%) occurring in the mandible and 21 cases (33%) in the maxilla. As for treatment, 26% of those with ONJ underwent antibiotic therapy, 18% were advised to use chlorhexidine mouthwashes, and surgery was opted for in 16% of the cases.

### 3.6. CDK4/6 Inhibitors

In the research conducted by Senin et al. [51], the association between the use of CDK4/6 inhibitors and the development of ONJ in patients undergoing treatment for breast cancer was explored. The study encompassed 243 patients, of which 95 (44.2%) were administered a CDK4/6 inhibitor. A total of 21 patients were diagnosed with MRONJ. Among these patients, 13 were treated with CDK4/6 inhibitors: 5 received abemaciclib, 5 were on palbociclib, and 3 were administered ribociclib. Notably, among all 19 patients treated with abemaciclib, there was a significantly increased risk of ONJ occurrence compared to those who did not receive CDK4/6 inhibitors (*p* = 0.0178). Marcianò et al. [52] described six cases of MRONJ associated with palbociclib (n = 5) and abemaciclib (n = 1) in patients treated for breast cancer. The authors linked the development of stomatitis/mucositis, a side effect of CDK4/6 inhibitors, to a potential risk for ONJ. Stomatitis involves the deterioration of the mouth’s mucosal lining, exposing the underlying bone to bacterial infection [51,52]. Yosofi et al. [53] presented a case series involving eight patients who underwent treatment with palbociclib. The authors did not identify any specific patterns, suggesting that palbociclib plays a triggering role in the onset of ONJ.

### 3.7. Other TKIs

Gürsoy [54] reported a case of avascular necrosis (AVN) of the femoral head following alectinib treatment for anaplastic lymphoma. Given the critical nature of the condition, the treatment proceeded with a careful evaluation of the benefits and risks.

Myoken et al. [55] described the case of a 65-year-old male with no prior history of antiresorptive therapy who developed ONJ after being treated with bosutinib for CML for one year. The treatment involved the extraction of the second premolar, which had a periapical lesion and the complete removal of the necrotic bone. This periapical lesion was associated with an osteolytic lesion. Infected soft tissue debridement was carried out, resulting in a medium-sized defect (2 cm × 4 cm) in the mandible. Due to the inadequacy of poorly vascularized mucoperiosteal tissue around the necrotic bone for stable wound closure and to prevent MRONJ relapse, a nasolabial flap was created to ensure sufficient well-vascularized soft tissue covered the decorticated bone, facilitating wound closure. The primary way bosutinib causes ONJ might be due to its harmful effects on osteoclasts and osteoblasts. This is because it blocks the tyrosine kinase Src/Abl pathway, leading to a disruption in bone remodeling [55].

Thekkudan and Nityanand [56] reported on a patient who developed AVN of the femoral head after nine months of nilotinib treatment for CML. A total hip replacement is planned for the patient. The authors suggested a mechanism that involves leukocytosis and/or thrombocytosis, leading to the occlusion of the microcirculation, or a combined effect from CML and INF-α treatment that results in the suppression of new blood vessel formation.

Axitinib acts as a selective blocker of VEGF receptor tyrosine kinases 1, 2, and 3 [57]. Patel et al. [57] documented a case involving a 67-year-old man who developed exposed necrotic bone in the right posterior maxilla following axitinib therapy for renal cell carcinoma. The patient was counseled to improve his oral hygiene with hydrogen peroxide mouthwash. He underwent bimonthly evaluations of the ONJ with conservative treatment. Axitinib’s ability to disrupt the angiogenic process is likely key to its involvement in causing ONJ.

Pazopanib is a multi-kinase inhibitor, effectively blocking angiogenesis and obstructing tumor expansion. Papadopoulou et al. [58] described three patients, aged 64, 63, and 56 years, who developed ONJ following pazopanib therapy for RCC. The treatment regimen for these patients included antibiotics and mouth rinses with 0.2% chlorhexidine. During follow-up visits, all patients showed no complaints or clinical indications of jaw osteonecrosis. The authors connected ONJ with the antiangiogenic properties of pazopanib, which result from the inhibition of VEGF.

Antonuzzo et al. [59] documented the case of a 59-year-old woman who, after 22 months of regorafenib treatment, presented with exposed necrotic bone in the upper jaw, accompanied by pain and soft tissue inflammation. It is noteworthy that the patient had previously been treated with antiangiogenic agents, like bevacizumab and aflibercept, which are known to be associated with ONJ [60].

Marino et al. [61] described the case of a 51-year-old female who experienced a non-healing socket in the left mandible three months following dental extraction due to deep caries. The patient was undergoing treatment with cabozantinib for thyroid carcinoma. Despite attempts at the surgical debridement of the socket and antibiotic therapy, no clinical improvement was observed. Consequently, a segmental ostectomy was carried out, which included the removal of the mandibular left second molar. The patient continued with antibiotic and antiseptic therapy, specifically oral amoxicillin clavulanate and chlorhexidine 0.2% mouthwash, until mucosal healing was achieved. ONJ after cabozantinib is associated with the VEGF pathway inhibition.

## 4. Immunotherapy

Immunotherapy using monoclonal antibodies has become a fundamental part of cancer treatment, complementing traditional approaches like surgery, radiation, and chemotherapy. These antibodies exhibit a wide array of clinically significant mechanisms and can directly attack tumor cells while also triggering anti-tumor immune responses [62]. Although monoclonal antibodies are highly effective in treating malignant tumors, their anti-angiogenic properties could elevate the risk of ONJ [10]. Table 3 provides detailed information on immunotherapy agents associated with osteonecrosis [62].

OpenVigil 2.1 is a platform developed for the analysis of adverse drug event data [63]. Li et al. [64] used this platform to gather raw data, aiming to identify cases of ONJ associated with treatment using immune checkpoint inhibitors. The researchers found 41 cases of ONJ related to the use of immune checkpoint inhibitors, due to atezolizumab (5 cases), ipilimumab (5 cases), nivolumab (26 cases), and pembrolizumab (5 cases). Male patients (16 cases) were more commonly affected than female patients (6 cases). Out of the reports that included age details, the age range of the patients was from 22 to 76 years. The conditions most frequently associated with these reports were metastatic renal cell carcinoma (seven cases) and NSCLC (five cases).

### 4.1. Bevacizumab

Bevacizumab, a monoclonal antibody targeting VEGF-A, inhibits angiogenesis. VEGF stimulates the growth and survival of endothelial cells and increases the permeability of vessels, meeting the tumor’s growing metabolic demands [65].

Numerous reports have documented instances of osteonecrosis following bevacizumab treatment. While the majority of these reports involve cases of ONJ, there are also instances of osteonecrosis occurring in the wrist and knee [66,67,68,69,70,71,72,73,74,75,76,77,78,79,80,81,82]. In a phase II clinical trial involving 30 patients who received bevacizumab for NSCLC, the incidence rate of ONJ was found to be 10% [73]. In the research conducted by Kose et al. [77], it was found that, out of 106 patients with recurring ovarian cancer who received bevacizumab treatment, 2 developed osteonecrosis. However, the study did not specify which bones were affected.

Fangusaro et al. [68] identified three pediatric cases of osteonecrosis linked to bevacizumab in patients undergoing treatment for brain tumors. The first case involved a 13-year-old girl who had never received steroids or bisphosphonates. The patient experienced tenderness and reduced movement in the left dorsal wrist. The MRI results showed extensive bone marrow edema and slight disintegration and collapse of the lunate bone, indicative of Kienbock’s disease. In the second case of a 17-year-old boy, the patient developed bilateral knee pain after five rounds of bevacizumab, with MRI scans suggesting early osteonecrosis in the distal femurs. The third patient, a 10-year-old girl, although showing no symptoms, had MRI findings of bilateral medullary bone infarcts in both knees, a sign consistent with osteonecrosis in the diametaphyses. Mir et al. [79] described three adult patients who developed AVN of the femoral head following bevacizumab treatment. The authors recommended that healthcare professionals should recognize that bone or joint pain in patients undergoing anti-VEGF therapy might stem not just from disease advancement, particularly in cancers that commonly spread to the bone, but also from bone complications related to the treatment.

Bevacizumab has the potential to compromise the integrity of microvessels in the jaw, potentially resulting in bone necrosis. Additionally, the critical role of VEGF in osteogenic differentiation and bone formation might account for this adverse effect [78,83]. Notably, the antiangiogenic effects of bevacizumab vary with the dosage and duration of treatment. This suggests that angiogenesis, along with bone remodeling and healing, may resume once the drug is discontinued. Bevacizumab’s estimated half-life is around 20 days, though it can range from 11 to 50 days [72,84]. Although the development of osteonecrosis during bevacizumab treatment is a rare side effect, it is important for patients to undergo careful evaluation.

### 4.2. Pembrolizumab

Patel and Carey [85] documented the case of a 73-year-old male who received a six-week course of pembrolizumab for melanoma and developed sustained exposed bone in the lower right mandible. A conservative strategy focusing on maintaining local hygiene and waiting for natural exfoliation was decided upon. In a separate report by Decaux and Magremanne [86], a 28-year-old woman undergoing treatment with pembrolizumab and epacadostat for melanoma experienced pain and bleeding around her first right maxillary molar following therapy. Due to the rapid progression of her metastatic condition, oral surgical intervention was not suggested. She persisted with mouth rinses and was administered morphine for pain management. Also, Pennings et al. [87] reported the case of a 44-year-old female with Hodgkin lymphoma who developed ONJ following treatment with pembrolizumab. The patient was recommended to maintain oral hygiene at home following the excision of the necrotic bone section. Four days later, she received an autologous stem cell transplant and reported no additional oral symptoms.

One potential explanation for the association between pembrolizumab and ONJ is its ability to reactivate T cells by blocking the binding of PD-1 to its ligands on the tumor site, thereby restoring the immune system. This T-cell activation may lead to the production of both pro-osteoclastogenic and anti-osteoclastogenic cytokines. Consequently, the occurrence of necrosis could be influenced by the activation of a specific subset of T cells that results in an elevation of anti-osteoclastogenic cytokines [88,89,90].

### 4.3. Ipilimumab

Ipilimumab, a monoclonal antibody, targets the CTLA4 receptor found on activated T lymphocytes. This interaction enhances the activity of T lymphocytes against melanoma cells, leading to their destruction. Owosho et al. [91] documented the first case of ONJ following the treatment of a patient, who was a 52-year-old male, with metastatic melanoma using ipilimumab. The patient was treated with a chlorhexidine rinse and, at the 2-month follow-up, the symptoms had fully resolved. Guida et al. [92] reported the case of a 58-year-old female who developed ONJ following ipilimumab treatment for metastatic melanoma. The osteonecrosis was treated with a combination of amoxicillin, metronidazole, and 0.2% chlorhexidine mouth rinse. At a follow-up visit two weeks later, the patient demonstrated clinical improvement. The patient referred the ejection of a 10 × 5 mm bone sequestrum after six days of therapy, resulting in the disappearance of the symptoms. The authors proposed that ipilimumab might contribute to bone necrosis by increasing the number of systemically activated T cells. It has been observed that CTLA4-deficient activated T cells are linked to osteonecrosis. This is because activated T cells can stimulate osteoclastogenesis through the osteoprotegerin ligand, leading to bone loss [92].

### 4.4. Nivolumab

Nivolumab acts on the PD-1 receptor found on activated T cells, thereby enhancing anti-tumor immune responses [93]. Pundole et al. [94] described the case of a 75-year-old male who developed jaw pain one week following their initial dose of nivolumab for metastatic melanoma. Although imaging tests showed no abnormalities and treatment was continued once the pain was managed, the patient suffered a severe increase in pain and jaw misalignment four months later. Subsequent imaging revealed bilateral fractures at the mandible’s angles along with a significant disruption of the normal trabecular structure, necessitating a total mandibulectomy. Nivolumab blocks inhibitory signals that prevent T-cell activation. Since T cells contribute to maintaining bone balance, this suggests that similar molecular pathways may play a role in the development of ONJ (pundole).

### 4.5. Trastuzumab

Trastuzumab is a humanized monoclonal antibody with antiangiogenic potential utilized for treating breast cancer in patients with an overexpression of the human epidermal growth factor receptor 2 (HER2) [95,96]. There are limited reports of ONJ following treatment with trastuzumab. In a study by Pilanci et al. [97], 97 breast cancer patients treated with zoledronic acid were examined, among whom 31 patients also received trastuzumab. Out of these patients, 13 developed ONJ. The study identified the length of zoledronic acid treatment and the administration of trastuzumab as two key factors contributing to the development of ONJ, with respective *p*-values of 0.049 and 0.028. Manzie et al. [98] reported the case of a 76-year-old female with ONJ linked to the use of pertuzumab and trastuzumab, which are used in treating HER2-positive metastatic breast cancer. The patient underwent sequestrectomy, and complete healing was observed nine months after the procedure. This case is particularly noteworthy as it occurred in a patient who had not previously been treated with any antiresorptive medications commonly associated with ONJ.

Trastuzumab inhibits angiogenesis, and this effect results from the induction of antiangiogenic factors and the suppression of proangiogenic factors. The combination of bisphosphonates with antiangiogenic agents, such as trastuzumab, may lead to a higher incidence of ONJ compared to the use of bisphosphonates alone [99].

### 4.6. Ramucirumab

Ramucirumab is a completely human monoclonal antibody belonging to the IgG1 class that blocks angiogenesis by attaching to the extracellular domain of VEGFR-2 [100]. Compared to bevacizumab, which targets VEGF-A and was the initial angiogenesis inhibitor used in clinical settings, ramucirumab inhibits the binding of various VEGF ligands. This variation in blocking different ligands could be associated with the development of delayed healing in dry sockets (IJIMA). Singh et al. [101] reported the case of a 59-year-old male patient, who was undergoing treatment with ramucirumab for NSCLC with brain metastasis and had not received any prior antiresorptive drug therapy. The patient exhibited spontaneous bone exposure in the right posterior lingual area of the mandible. He was advised to use a 0.12% chlorhexidine gluconate oral rinse twice a day. Iijima et al. [102] reported the case of a 76-year old male with gastric cancer experiencing delayed dry socket healing attributed to ramucirumab treatment. Following the tooth extractions, the patient was treated with amoxicillin for 7 days and used acetaminophen for pain relief. While the healing of the tooth sockets was ultimately successful, it required approximately 150 days. It is important for dentists and oral surgeons to recognize that inhibitors of angiogenesis can lead to conditions such as ONJ and dry sockets following surgical intervention. 

## 5. Conclusions

The portfolio of anti-cancer treatment has undergone a significant transformation in recent years, offering patients targeted therapies that substantially extend overall survival. However, treatments with TKIs and immunotherapies are not without side effects. A notable adverse effect is osteonecrosis, which is most commonly found in the jaw but can also occur in other bones, such as the lunate and femur. Despite the relatively low number of reports on osteonecrosis following novel anti-cancer treatments, primarily consisting of single case reports, it is crucial to carefully monitor the occurrence of such adverse events in both physicians’ practice and clinical trials.

Since there are no specific recommendations for patients and physicians regarding the management of osteonecrosis after novel anti-cancer treatment, some basic risk reduction measures can be implemented. Patients should be informed about the potential risk of osteonecrosis associated with their treatment. They should also be advised on the importance of maintaining good oral hygiene and having regular dental check-ups. It is crucial for the treating physician to remain vigilant and informed about these potential complications, especially since ONJ lacks a specific treatment and prevention and early diagnosis is crucial. To achieve the best outcomes, conducting a thorough risk assessment before and throughout the treatment process is essential. Additionally, patients may be advised to avoid elective dental extractions and other surgical dental procedures while undergoing therapy. If such procedures are necessary, it is advisable to consult with the patient’s oncologist to assess the risk and plan appropriately.

## Figures and Tables

**Table 1:** Classification and treatment of medication-related osteonecrosis of the jaw [5].

Stage	Symptoms and Findings	Management
0	No clinical signs of necrotic bone present; however, nonspecific clinical observations, changes visible in radiographs, and symptoms are noted.	Systemic antibiotics and painkillers.
I	Patients are asymptomatic and show no signs of infection, but have exposed and necrotic bone or fistulas that probe to bone. May also display radiographic findings like those described for Stage 0, localized specifically to the region of the alveolar bone.	Antibacterial mouth rinse, follow-up every four months, educating the patient, and evaluating the ongoing need for bisphosphonate therapy.
II	Exposed and necrotic bone, or fistulas probing to bone, coupled with infection, as indicated by pain and redness in the exposed bone, with or without the presence of pus drainage. May also display radiographic findings similar to those described for Stage 0, localized specifically to the region of the alveolar bone.	Treatment focused on relieving symptoms includes oral antibiotics, antibacterial mouth rinses for oral hygiene, managing pain, and debridement to alleviate the irritation of the soft tissue and manage infection.
III	Patients with exposed and necrotic bone or a fistula that probes to bone, experiencing pain and infection, alongside one or more of the following findings: Necrotic bone exposure beyond the alveolar bone, including the mandible’s inferior border and ramus, and the maxilla’s maxillary sinus and zygoma. Pathological fracture. Extraoral fistula. Communication between the oral cavity and nasal sinus or antrum. Osteolysis reaching the mandible’s inferior border or sinus floor.	Use of antibacterial mouth rinse, antibiotic treatment and pain management, followed by surgical debridement or resection to provide long-term relief from infection and pain.

**Table 2:** Characteristics of TKIs associated with osteonecrosis [18,19].

Agent	Target	Inhibitor Class	Indications	Level of Evidence
Abemaciclib	CDK4/6	I	HR+ and HER2− breast cancers	Retrospective study Case series
Alectinib	ALK, RET	II	ALK+ NSCLC	Case reports
Axitinib	PDGFRβ, VEGFR1/2/3	II	RCC	Case reports
Bosutinib	BCR-ABL, Src, Lyn, Hck	I	CML	Case reports
Cabozantinib	RET, Met, VEGFR1/2/3, Kit, TrkB, Flt3, Axl, Tie2, ROS1	I	Metastatic medullary thyroid cancer RCC HCC	Case reports
Imatinib	BCR-ABL, PDGFR, c-kit	II	CML ALL GIST Dermatofibrosarcoma protuberans Myeloproliferative and myelodysplastic syndromes	Case reports
Lenvatinib	VEGFRs, FGFRs, PDGFR, Kit, RET	II	Differentiated thyroid carcinoma	Case reports
Nilotinib	BCR-ABL, PDGFR, DDR1	II	Ph+ CML, CLL, ALL	Case reports
Osimertinib	EGFR, T970M	Covalent (V)	NSCLC	Case reports
Palbociclib	CDK4/6	I	ER+ and HER2− breast cancers	Retrospective study Case series Case reports
Pazopanib	VEGFR1/2/3, PDGFRα/β, FGFR1/3, Kit, Lck, Fms, Itk	I	RCC STS	Case series
Ribociclib	CDK4/6	I	HR+ and EGFR− metastatic breast cancers	Retrospective study
Regorafenib	VEGFR1/2/3, BCR-ABL, BRAF, BRAF(V600E), Kit, PDGFRα/β, RET, FGFR1/2, Tie2, Eph2A	II	CRC GIST	Case reports
Sorafenib	B/C-Raf, BRAF (V600E), Kit, Flt3, RET, VEGFR1/2/3, PDGFRβ	II	RCC DTC HCC	Case reports
Sunitinib	PDGFRα/β, VEGFR1/2/3, c-Kit, Flt3, CSF-1R, RET	II	RCC GIST GIST	Systematic review Case reports

ALK—anaplastic lymphoma kinase; ALL—acute lymphoid leukemia; BCR-ABL—breakpoint cluster region-Abelson; CDK—cyclin-dependent kinase; CLL—chronic lymphoid leukemia; CML—chronic myeloid leukemia; CRC—colorectal carcinoma; CSF-1R—colony-stimulating factor-1 receptor; DTC—differentiated thyroid carcinoma; EGFR—epidermal growth factor receptor; FGFR—fibroblast growth factor receptor; GIST—gastrointestinal stromal tumor; HCC—hepatocellular carcinoma; HER—human epidermal growth factor receptor; HR—hormone receptor; NSCLC—non-small cell lung cancer; Ph+—Philadelphia chromosome; PDGFR—platelet-derived growth factor receptors; RCC—renal cell carcinoma; RET—rearranged during transfection; STS—soft tissue sarcoma; VEGFR—vascular endothelial growth factor receptor.

**Table 3:** Characteristics of immunotherapy agents associated with osteonecrosis.

Agent	Format	Molecular Target	Indications	Level of Evidence
Bevacizumab	Humanized IgG1	VEGF ligand	Colorectal cancer, NSCLC, RCC, glioblastoma, ovarian cancer	Clinical randomized trial Case series Case reports
Pembrolizumab	Humanized IgG1	PD-1	Melanoma, various cancers	Case reports
Ipilimumab	Human IgG1	CTLA-4	Melanoma, RCC	Case reports
Nivolumab	Human IgG1	PD-1	Melanoma, lung cancer, RCC	Case reports
Trastuzumab	Humanized IgG1	HER2	Breast cancer	Retrospective study Case reports
Pertuzumab	Humanized IgG1	HER2	Breast cancer	Case reports
Ramucirumab	Human IgG1	VEGFR2	Gastric cancer, NSCLC	Case reports

CTLA—cytotoxic T-lymphocyte-associated antigen; HER—human epidermal growth factor receptor; NSCLC—non-small cell lung cancer; PD—programed cell death; RCC—renal cell carcinoma; VEGF—vascular endothelial growth factor; VEGFR—vascular endothelial growth factor receptor.

## Data Availability

Not applicable.
